# Effects of SGLT2 inhibition on incident heart failure in carriers of cardiomyopathy-associated genetic variants

**DOI:** 10.1038/s41591-026-04439-x

**Published:** 2026-06-08

**Authors:** Nicholas A. Marston, Shinwan Kany, Giorgio E. M. Melloni, Sean J. Jurgens, Frederick K. Kamanu, Yi-Pin Lai, Joel T. Rämö, Itamar Raz, Stephen D. Wiviott, Patrick T. Ellinor, Marc S. Sabatine, Christian T. Ruff

**Affiliations:** 1 Division of Cardiovascular Medicine, Brigham and Women’s Hospital, Boston, MA USA; 2 Thrombolysis in Myocardial Infarction Study Group, Brigham and Women’s Hospital, Boston, MA USA; 3 Harvard Medical School, Boston, MA USA; 4 Department of Cardiology, University Heart and Vascular Center Hamburg-Eppendorf, Hamburg, Germany; 5 German Center for Cardiovascular Research (DZHK), Partner Site North, Hamburg, Germany; 6 Cardiovascular Disease Initiative, Broad Institute of MIT and Harvard, Cambridge, MA USA; 7 Department of Experimental Cardiology, Amsterdam Cardiovascular Sciences, Heart Failure and Arrhythmias, Amsterdam UMC, location University of Amsterdam, Amsterdam, the Netherlands; 8 Department of Medicine, Brigham and Women’s Hospital, Boston, MA USA; 9 Department of Endocrinology and Metabolism, Hadassah Hebrew University Hospital, Jerusalem, Israel; 10 Late-Stage Development, Cardiovascular, Renal, and Metabolism BioPharmaceuticals R&D, AstraZeneca, Boston, MA USA; 11 Heart and Vascular Institute, Mass General Brigham, Boston, MA USA

**Keywords:** Cardiovascular diseases, Genetics research

## Abstract

Although the beneficial effects of sodium–glucose cotransporter 2 (SGLT2) inhibition in heart failure (HF) have been well established, it is unknown whether SGLT2 inhibition confers benefit in carriers of rare variants in cardiomyopathy-associated genes. Here we evaluated whole-exome sequencing data from the randomized DECLARE-TIMI 58 trial, in which adults with type 2 diabetes and increased cardiovascular risk were randomized to dapagliflozin or placebo treatment. Pathogenic or likely pathogenic variants (P/LP) in high-confidence cardiomyopathy genes were identified, and treatment effects on hospitalization for HF (HHF) were compared between carriers of such variants and noncarriers. Among 12,685 patients for whom sequence data were obtained, 121 carried a cardiomyopathy variant (76 dilated cardiomyopathy, 25 hypertrophic cardiomyopathy and 25 arrhythmogenic cardiomyopathy). Over a median follow-up of 4.2 years, dapagliflozin lowered the risk of HHF more strongly in carriers (hazard ratio 0.18, 95% confidence interval 0.04–0.86) than in noncarriers (hazard ratio 0.70, 95% confidence interval 0.57–0.86; *P* interaction 0.03). Absolute risk reduction was 13.0% in carriers and 1.0% in noncarriers (*P* interaction 0.03). Most carriers (82%) had no prior HF, and in carriers without prior HF, treatment with dapagliflozin reduced the absolute risk of HHF by 12.8%, compared with a reduction of 0.6% in noncarriers (*P* interaction 0.01). The findings from this cohort of older and high-risk patients raise the possibility that SGLT2 inhibitor treatment should be started early to prevent HF in individuals who carry P/LP cardiomyopathy variants. These results need to be confirmed in a prospective, dedicated trial of preventive HF treatments in carriers of P/LP cardiomyopathy-associated variants.

## Abstract (web summary)

In a whole-exome sequencing analysis, the beneficial effects of the SGLT2 inhibitor dapagliflozin in reducing the risk of future heart failure hospitalization in individuals with type 2 diabetes were markedly greater in individuals who carried a cardiomyopathy-associated genetic variant compared with noncarriers, suggesting a personalized preventative therapy based on genetic information.

## Main

Heart failure (HF) is associated with substantial morbidity and mortality^1^. Sodium–glucose cotransporter 2 (SGLT2) inhibitors such as dapagliflozin have been shown to prevent hospitalization for HF (HHF) across the spectrum of left ventricular ejection fraction (LVEF)^2–4^. In the Dapagliflozin Effect on Cardiovascular Events – Thrombolysis in Myocardial Infarction 58 (DECLARE-TIMI 58) trial, dapagliflozin reduced the incidence of HHF in patients with type 2 diabetes with, or at high risk for, atherosclerotic cardiovascular (CV) disease^2^.

Inherited cardiomyopathies (CMPs) are an increasingly recognized contributing factor to HF. These conditions reflect a genetic predisposition to structural and rhythm abnormalities of the heart leading to increased risk for HF and death^5^. Due to the considerable heritability in genetic forms of CMP, genetic testing is an essential step in the diagnostic pathway of these diseases as outlined in European and American professional guidelines^6,7^. Pathogenic or likely pathogenic (P/LP) variants are commonly observed in patients with CMP. In addition, CMP variants have an estimated prevalence of ~1% in the general population, including asymptomatic individuals who are at high genetic risk of incident CMP in the future^8–12^.

Apart from increased surveillance and monitoring, the therapeutic consequence of identifying CMP variants, particularly in asymptomatic individuals, remains unclear^6^. Such situations are increasingly frequent due to cascade screening of family members of patients with CMP as well as incidental findings from genomic sequencing conducted for other indications. Whether SGLT2 inhibitor treatment is of benefit in asymptomatic individuals with a CMP variant is not known.

In this analysis, we tested the effect of the SGLT2 inhibitor dapagliflozin compared with placebo among carriers of CMP-associated genetic variants and noncarriers in the DECLARE-TIMI 58 trial.

## Results

### Baseline characteristics and prevalence of CMP variants

A total of 12,685 patients were eligible for the genetic substudy, with 121 (1%) carrying at least one CMP-associated variant (Table 1). The patients were not meaningfully different from the overall trial population (Supplementary Table 1). Within carriers, and not mutually exclusive, 76 carried dilated CMP (DCM) variants, 25 hypertrophic CMP (HCM) variants and 25 arrhythmogenic right ventricular CMP (ARVC) variants. The most common gene mutation was titin (*TTN*), with 57 patients having a P/LP variant in cardiac exons of the *TTN* gene, followed by 8 carriers each of *MYH7* and *PKP2*. The full variant by gene count is provided in Table 2. Out of the 121 CMP variant carriers, 65 received dapagliflozin and 56 received placebo (Extended Data Table 1). Baseline characteristics were broadly similar between carriers and noncarriers, although carriers more often had prior HF (18.2% versus 9.8%, *P* = 0.004) and usage of mineralocorticoid receptor antagonist (MRA) therapy (12.4% versus 4.7%, *P* < 0.001). MRA therapy was more utilized in carriers of CMP-associated variants with a history of HF than those without (40.9% versus 6.1%, *P* < 0.001) (Extended Data Table 2). Similar observations were made when stratifying noncarriers by a history of HF (Extended Data Table 2). Among participants with LVEF available, carriers had lower LVEF (51% versus 56%, *P* = 0.002) (Table 1). N-terminal pro-brain natriuretic peptide (NT-proBNP) levels were also higher in carriers of CMP-associated variants versus noncarriers (299.6 ± 625.2 pg ml^−1^ versus 171 ± 321.4 pg ml^−1^, *P* < 0.001) while high-sensitivity cardiac troponin T (hs-TnT; 13.6 ± 9.6 ng l^−1^ versus 13.1 ± 15.5 ng l^−1^, *P* = 0.49) was not (Table 1). There was also a significant difference in NT-proBNP levels among carriers of CMP-associated variants between those with a history of HF versus those without a history of HF (629 ± 722.5 pg ml^−1^ versus 234.4 ± 586.6 pg ml^−1^, *P* < 0.001) (Extended Data Table 2).

**Table 1:** Baseline characteristics of the whole-exome population in the DECLARE-TIMI 58 trial

Characteristic	CMP P/LP variant carriers (*N* = 121)	Noncarriers (*N* = 12,564)	*P* value
Age	63.4 ± 6.4	64 ± 6.9	0.43
Male sex	74 (61)	8,051 (64)	0.57
Ethnicity	Asian 7 (6)	Asian 1,140 (9)	0.01
	Black 1 (1)	Black 396 (3)	
	Other 10 (8)	Other 442 (4)	
	White 103 (85)	White 10,586 (84)	
Body mass index, kg m^−^^2^	32.1 ± 5.5	32.5 ± 6	0.72
ASCVD	50 (41)	5,265 (42)	0.97
Prior myocardial infarction	23 (19)	2,765 (22)	0.49
History of hypertension	109 (90)	11,286 (90)	1.00
History of HF	22 (18)	1,235 (10)	0.004
History of diabetes	118 (98)	12,336 (98)	0.84
History of chronic kidney disease	11 (9)	944 (8)	0.63
History of atrial fibrillation	14 (12)	867 (7)	0.07
Current smoker	16 (13)	1,770 (14)	0.89
Systolic blood pressure, mm Hg	133.8 ± 16.5	135.1 ± 15.3	0.59
eGFR, ml min^−1^ 1.73 m^−^^2^	87.7 ± 22.7	85.9 ± 21.7	0.34
NT-proBNP, pg ml^−1^	299.6 ± 625.2	171 ± 321.4	0.0004
hs-TnT, ng l^−1^	13.6 ± 9.6	13.1 ± 15.5	0.49
MRA therapy	15 (12.4%)	592 (4.7%)	0.0002
RAAS inhibitor therapy	98 (81%)	10,382 (82.6%)	0.72
LVEF (%) (*N* = 3,247)	50.8 ± 11.9	56.5 ± 11.1	0.002
LVEF <50%	12/36 (33.3%)	667/3,211 (20.8%)	0.10
LVEF <40%	7/36 (19.4%)	240/3,211 (7.5%)	0.02

Continuous variables are reported as mean ± s.d. and tested with a two-sided Wilcoxon rank-sum test. Categorical variables are reported as *n* (%) and tested with a two-sided chi-squared test.

Baseline characteristics of CMP variant carriers versus noncarriers in the genetic cohort of DECLARE-TIMI 58. Presented as counts (percentage) or mean ± s.d. eGFR, estimated glomerular filtration rate; RAAS, renin–angiotensin–aldosterone system.

**Table 2:** Variant carrier count by genes

Gene	DCM *N* = 76	HCM *N* = 25	ARVC *N* = 25	CMP *N* = 121
*ACTC1*		0		0
*ACTN2*		0		0
*BAG3*	1			1
*CSRP3*		0		0
*DES*	0			0
*DSC2*			5	5
*DSG2*			7	7
*DSP*	5		5	5
*FLNC*	4			4
*LMNA*	1			2
*MYBPC3*		6		6
*MYH7*	1	8		8
*MYL2*		0		0
*MYL3*		0		0
*PKP2*			8	8
*PLN*	6	6		6
*PRKAG2*		0		0
*RBM20*	0			0
*SCN5A*	0			4
*TMEM43*			0	0
*TNNC1*	0	0		0
*TNNI3*		3		4
*TNNT2*	1	1		1
*TPM1*		1		1
*TTN*	57			57
*TTR*		0		3

The CMP column includes all P/LP variants in the curated gene list irrespective of the specific phenotype reported in ClinVar. Consequently, variants in genes without a specific phenotype report in ClinVar are included in the overall CMP count but excluded from the disease-specific subcolumns (DCM, HCM and ARVC). Genes associated with multiple CMP phenotypes (for example, *DSP* and *PLN*) are counted in all relevant subphenotype columns, resulting in row sums that may exceed the CMP total. One individual carried P/LP variants in two genes.

### Association of carrier status with incident outcomes in the placebo arm

**Fig. 1:**
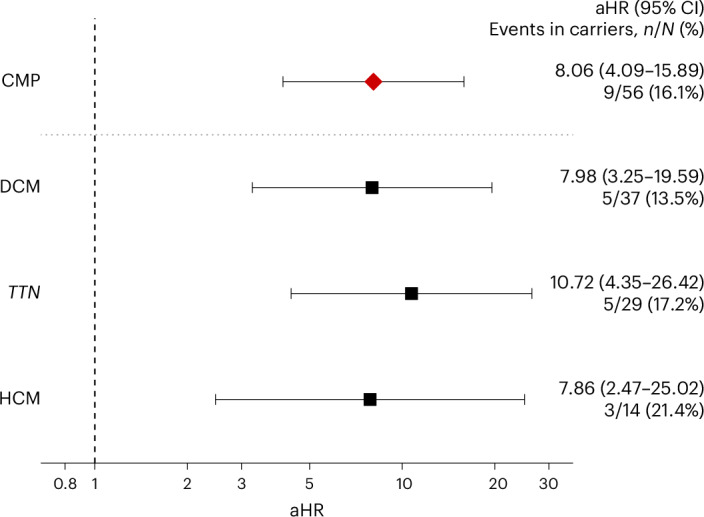
Associations of variant carrier status with incident outcomes. Cox proportional hazard models were used to evaluate the association of variant carrier category with incident HHF in the placebo arm. Models were adjusted for age, sex, body mass index, current smokers at baseline, systolic blood pressure, history of coronary artery disease, history of myocardial infarction and estimated glomerular filtration rate and the principal components 1–10 for genetic ancestry. The reference group was the noncarrier group. Data are presented as point estimates (aHRs), with error bars indicating the 95% CIs. *P* values were obtained by two-sided Wald test. *n*/*N*, events/total carriers. Data for ARVC are not shown due to the lack of events in 25 carriers for such variants.

To establish the prognostic value of carrying a rare variant in a CMP-associated gene, we tested the association between carrier status and HHF in the placebo group. In the total genetic cohort of DECLARE-TIMI 58, 389 individuals had a HHF event at the end of the median 4.2-year follow-up period. In the placebo arm, the incidence of HHF was 3.5% (221/6,291) in noncarriers and 16% (9/56) in carriers of CMP-associated variants, translating to an over eightfold increased relative risk among carriers of CMP-associated variants (adjusted hazard ratio (aHR) 8.06, 95% confidence interval (CI) 4.09–15.89, *P* < 0.001). This markedly increased risk was consistent across carriers of DCM variants (aHR 7.98, 95% CI 3.25–19.59, *P* < 0.001), carriers of *TTN* variants (hazard ratio (HR) 10.72, 95% CI 4.35–26.42, *P* < 0.001), and carriers of HCM variants (aHR 7.89, 95% CI 2.48–25.10, *P* < 0.001) (Fig. 1). Even after further adjusting for baseline NT-proBNP levels, carriers of CMP-associated variants were at sevenfold increased relative risk compared with noncarriers (aHR 7.35, 95% CI 3.67–14.73, *P* < 0.0001). Secondary exploratory endpoints included the composite of CV death or HHF, isolated CV death and all-cause mortality and were not statistically different between carriers of CMP-associated variants and noncarriers (Supplementary Table 2).

### Treatment effect by carrier status

Then, we sought to establish the treatment effect of dapagliflozin in carriers of CMP-associated variants carriers versus noncarriers.

**Fig. 2:**
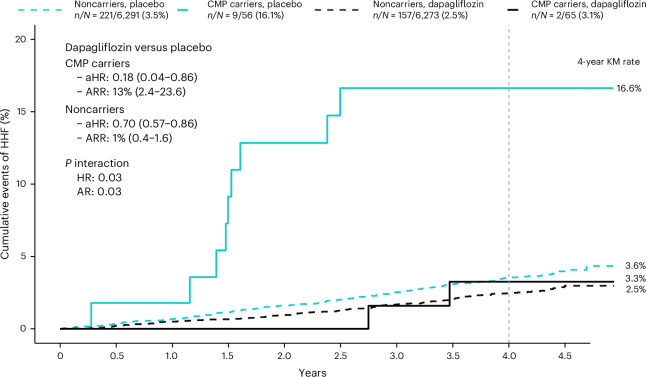
Efficacy of dapagliflozin for HF hospitalization by carrier status. Kaplan–Meier (KM) cumulative incidence curves are shown for HHF in carriers of CMP-associated variants (solid lines) and noncarriers (dashed lines). The teal lines represent the placebo group, and the black lines represent the dapagliflozin group. The *y* axis depicts the cumulative event rate over the follow-up period (median 4.2 years). Interaction *P* values obtained by two-sided Wald test on a Cox proportional hazard model (HR) and a generalized linear model using pseudo-values (absolute risk (AR)).

In the overall genetic cohort, treatment with dapagliflozin led to a 32% reduction in the incidence of HHF (HR 0.68, 95% CI 0.55–0.83, *P* < 0.001) compared with placebo. Among CMP variant carriers, treatment with dapagliflozin resulted in an 82% reduction in incident HHF (HR 0.18, 95% CI 0.04–0.86). This was significantly greater than the 30% risk reduction observed in noncarriers (HR 0.70, 95% CI 0.57–0.86, *P* interaction 0.03) (Fig. 2). The use of dapagliflozin among patients with CMP-associated variants decreased the incidence of HHF over a median 4.2 years from 16.1% to 3.1% (Kaplan–Meier rate 16% and 3.6%, respectively), achieving the risk level observed in noncarriers. This 13.0% (2.4–23.6%) absolute risk reduction (ARR) was significantly greater than the 1.0% (0.4–1.6%) ARR observed in noncarriers (*P* interaction 0.03) and translated to a number needed to treat of just 7.7 (compared with 100 in noncarriers) over 4.2 years (Fig. 2). These results were largely consistent across DCM and HCM variants (Supplementary Table 3) and held true even when adjusted for history of prior myocardial infarction and baseline NT-proBNP values (*P* interaction 0.009; Supplementary Table 4).

### Effect of dapagliflozin by history of HF

**Fig. 3:**
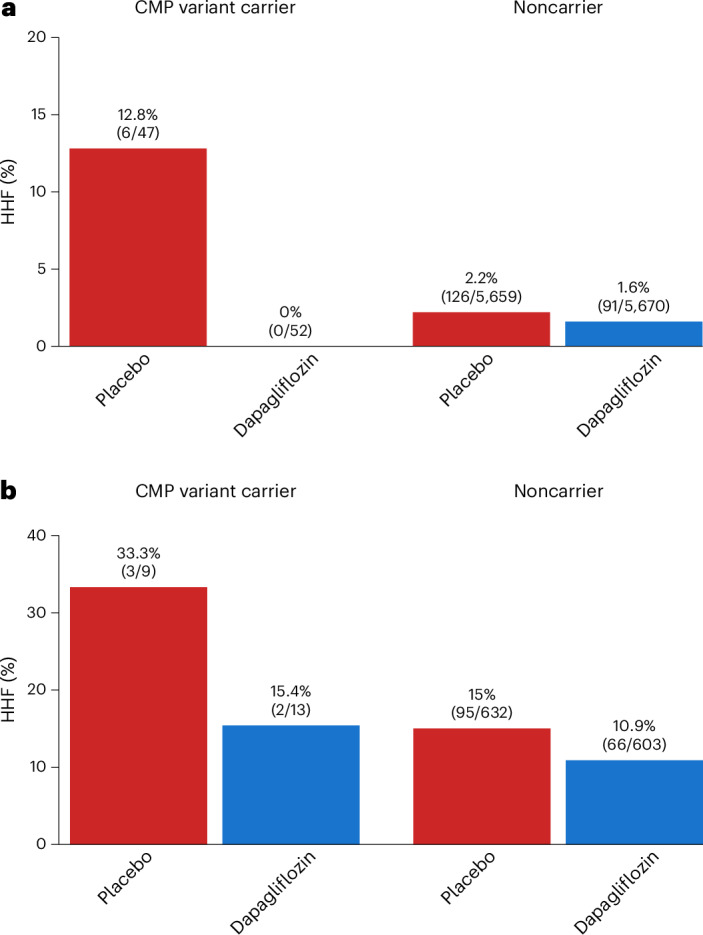
Efficacy of dapagliflozin for HF hospitalization by carrier status, as stratified by history of HF. **a**, The proportion of patients hospitalized for HF is shown for those without a previous history of HF, stratified by CMP variant carrier status. The HR for noncarriers was 0.72 (95% CI 0.55–0.94), whereas the HR for carriers of CMP-associated variants could not be calculated due to the lack of events in the dapagliflozin arm. The ARR was 12.8% (95% CI 3.1–22.4%) in CMP variant carriers versus 0.6% (95% CI 0.1–1.1%) in noncarriers (*P* interaction 0.01, from a generalized linear model using pseudo-values). **b**, The proportion of patients hospitalized for HF is shown for those with a history of HF, stratified by CMP variant carrier status. HRs were 0.67 (95% CI 0.49–0.92) and 0.60 (95% CI 0.10–3.78) in noncarriers and carriers, respectively. The ARR was 17.9% (95% CI −20.6% to 56.5%) in carriers of CMP-associated variants versus 4.1% (95% CI 0.3–7.8%) in noncarriers (*P* interaction 0.48). Treatment groups are indicated on the *x* axis (placebo group in red, dapagliflozin in blue), and the raw event proportion at the end of follow-up (median 4.2 years) is indicated on the *y* axis.

Most carriers of CMP-associated variants had no history of HF (82%, 99/121) (Extended Data Table 2). In the placebo arm, 12.8% of carriers without a history of HF developed HF during the trial. This represented an eightfold increase in the incidence of HHF (HR 8.23, 95% CI 3.61–18.79, *P* < 0.001) compared with noncarriers. This high risk of incident HF among carriers was mitigated with dapagliflozin treatment, with none of the patients allocated to dapagliflozin experiencing incident HF. This 12.8% ARR was significantly greater than what was observed in noncarriers without HF (ARR 12.8% (95% CI 3.1–22.4%) versus 0.6% (95% CI 0.1–1.1%), *P* interaction 0.01) (Fig. 3a and Supplementary Table 5). While carriers of CMP-associated variants with a history of HF had a numerically higher risk compared with noncarriers, this did not reach statistical significance (HR 2.98, 95% CI 0.88–10.20; *P* = 0.08). The HRs with dapagliflozin were 0.67 (95% CI 0.49–0.92) in noncarriers and 0.60 (95% CI 0.10–3.78) in carriers. The absolute risk differences were 4.1% (95% CI 0.3–7.8%) in noncarriers and 17.9% (95% CI 20.6–56.5%) in carriers (Fig. 3b and Supplementary Table 6).

## Discussion

In this study, we analyzed whole exomes in 12,685 participants from the DECLARE-TIMI 58 trial and identified 121 carriers of CMP-associated variants. We observed that patients with CMP variants in the placebo arm had an eightfold increased risk for incident HHF. Treatment with dapagliflozin reduced the incidence of HHF in both carriers and noncarriers, but we observed greater benefit among carriers of CMP-associated variants. Specifically, carriers derived a two- to threefold greater relative risk reduction and a tenfold greater ARR with dapagliflozin compared with the effect in noncarriers. In addition, carriers of CMP-associated variants without baseline HF experienced a particularly pronounced protective effect, suggesting that the early initiation of SGLT2 inhibitors may help delay or prevent HF in this high-risk group.

The prognostic significance of CMP variants has been well established through both disease-based cohorts as well as population-based studies. For example, in a Spanish cohort of 1,005 patients with DCM, the presence of a P/LP rare variant was associated with increased risk for HF^13^. Data from the population-based UK Biobank revealed a CMP variant carrier prevalence of 2.6%, with most carriers (95%) showing no clinical manifestation of HF at baseline^14^. Yet, these variant carriers were almost six times more likely to receive a CMP diagnosis in the future compared with noncarriers. Our work extends these findings to a clinical trial cohort, where we observed a carrier prevalence of 1%, with most (80%) being free of HF at baseline yet carrying significantly increased risk for incident HHF in the years to follow. We observed this despite the advanced age (mean 63 years) of carriers of CMP-associated variants.

Current US and European guidelines recommend genetic testing primarily when a genetic cause of CMP is strongly suspected or to facilitate cascade screening among relatives. Owing to the lack of large, dedicated clinical trials assessing the impact of rare CMP variants on treatment benefit, the primary motivation for genetic testing is risk stratification, early consideration of implantable cardioverter defibrillator devices^6,7^ or identification of variant carriers through family cascade screening. However, as shown in population-based studies^8,14^ and healthcare-based cohorts^15^, the majority of carriers of CMP-associated variants are asymptomatic at the time of their genetic diagnosis, leading to uncertainty about what can be done to prevent the development of the associated phenotype.

In this study, we demonstrate that CMP variants are actionable genotypes, in which therapeutic management may be influenced by the results of genetic testing. Not only does the presence of a CMP variant carry up to an eightfold greater risk of an HF event in those treated with placebo, but it also identifies patients who derive the greatest benefit from therapy. This is true for both the relative risk reduction, with an 82% decrease in HHF, and an absolute reduction of 13% over a median of 4.2 years. As a result, the number needed to treat to prevent a HF event is an order of magnitude lower.

What is perhaps most encouraging is that this treatment benefit seems to be greater among individuals without HF at baseline which raises the possibility that SGLT2 inhibition could be an effective HF prevention strategy in patients with a CMP variant and no clinical HF or known CMP. A recent study of carriers of DCM variants identified through cascade screening reported that 11% went on to develop DCM after initial negative evaluations, further highlighting the potential for preventive therapy in this high-risk group^16^. Taken together, these preliminary findings suggest that identifying a CMP genotype may help highlight individuals at elevated risk for incident HF. While currently hypothesis-generating, these data outline a potential future framework in which genetic testing could help inform the use of targeted preventive HF therapies.

The evidence for a preventive strategy in HF is growing, and initial safety trials such as DECLARE-TIMI 58 have helped to demonstrate the benefit of SGLT2 inhibitors for incident HF among patients with diabetes. More recently, the Prevent-HF trial (Phase III Study Investigating Heart Failure and Cardiovascular Death with Baxdrostat in combination with Dapagliflozin, NCT06677060) is actively studying dapagliflozin + baxdrostat versus dapagliflozin alone in patients with diabetes without prior HF with the aim of preventing incident HF. As we move into the era of preventive therapies for HF, more trials will need to be performed in patients without diabetes. In the SELECT trial, patients without diabetes but with increased body weight and atherosclerotic CV disease (ASCVD) had a significantly reduced risk of HF with semaglutide^17^. Recently, a scientific statement of the American Heart Association outlined the potential for preventive HF therapies and highlighted asymptomatic CMP variant carriers as an at-risk group for screening and risk factor modification^18^. Indeed, a recent study on 3,158 people with *TTN*tv-related DCM showed that those receiving beta-blocker therapy or renin–angiotensin system blocking therapy were less likely to develop overt DCM. However, these data were observational in nature and did not include systematic adjudicated events^19^.

Our results suggest that genetic information should be considered alongside, rather than in place of, established clinical screening strategies and biomarkers recommended by current guidelines. Clinical assessment, imaging when indicated, and circulating biomarkers remain central to the evaluation of CMP and HF risk. In this analysis, adjustment for baseline NT-proBNP did not materially alter the association between CMP variant carrier status and HF outcomes, which indicates that genetic risk captures information not fully reflected by biomarkers alone. Even so, inherited CMP variants do not replace phenotypic assessment, and their role will require prospective evaluation together with guideline-directed screening and management.

These findings must be interpreted in the context of our study design. Although this randomized dataset enabled evaluation of the interaction between rare CMP variant carrier status and SGLT2 inhibitor therapy, the relatively small number of carriers (*n* = 121) limits power for subgroup analyses by specific gene or morphologic subtype. Furthermore, as this was a post-hoc analysis of a trial in type 2 diabetes, the clinical presentation of these carriers probably reflects a composite of genetic susceptibility and metabolic comorbidities and might potentially favor a HF phenotype over the arrhythmic presentations often seen in younger, isolated CMP cohorts. Future prospective trials in variant carriers will require systematic screening for prior structural heart disease and granular adjudication of arrhythmia versus HF precipitants.

Another important limitation of this analysis is the absence of systematic cardiac imaging. DECLARE-TIMI 58 did not include protocolized echocardiography or cardiac magnetic resonance imaging, which precluded assessment of subclinical structural or functional CMP among carriers of genetic variants. Consequently, we are unable to determine whether the observed differences in HF outcomes reflect genetically mediated risk in the absence of overt disease or early but unrecognized cardiomyopathic changes, or progression of established myocardial abnormalities. Integration of standardized imaging in future studies will be essential to distinguish genotype-associated risk from phenotype-defined disease and to clarify the stage of CMP at which SGLT2 inhibition may exert the greatest benefit. In addition, as the trial enrolled patients with type 2 diabetes and high risk for CV disease, our findings may not be generalizable to populations without diabetes or lower CV risk. We also note that follow-up in this analysis was relatively short (median 4.2 years), and the long-term trajectory of HF risk in carriers of CMP-associated variants may differ over extended periods. Finally, as only 14% of participants were of non-European descent, our results may not apply to all ancestries.

In patients with type 2 diabetes mellitus and increased CV risk, carriers of CMP-associated variants experience a comparatively greater reduction in incident HF when treated with dapagliflozin. These findings were especially pronounced in individuals without prior HF and may inform evaluation of earlier initiation of SGLT2 inhibitor therapies that can prevent or delay the development of HF.

## Methods

### Study design

This is a secondary analysis of DECLARE-TIMI 58, a double-blind, randomized, placebo-controlled trial that investigated dapagliflozin in 17,160 patients with type 2 diabetes who had or were at risk for ASCVD. In this study, we investigate the effect of dapagliflozin among carriers and noncarriers of rare CMP variants in the subsample of DECLARE-TIMI 58 with available whole-exome sequencing data. This analysis was not prespecified.

One of the primary efficacy outcomes of the overall trial was a composite of CV death and HHF, which was significantly reduced by treatment with dapagliflozin compared with placebo (4.9% versus 5.8%, HR 0.83; 95% CI 0.73–0.95, *P* = 0.005)^2^. We focused on HHF as the primary endpoint of interest because in DECLARE-TIMI 58 the most robust treatment effects were observed for HHF, and HF is the outcome most directly related to CMP phenotypes. We also assessed secondary exploratory endpoints of CV death, a composite of CV death and HHF, as well as all-cause death. Patients who consented to genetic testing and had whole-exome sequencing available were evaluated for the presence of a CMP variant, defined as P/LP variants for DCM, HCM or ARVC based on stringent filtering criteria using annotation and ClinVar.

The trial protocol was approved by the institutional review board at each participating site, and all participants provided written informed consent.

### Sequence data quality control

Exome sequencing for 13,184 individuals was performed at the Broad Institute of MIT and Harvard on Illumina HiSeq 2500 using a TruSeq Rapid Exome Library prep kit. Sequence reads were aligned to GRCh38 with BWA 0.7.15-r1140 and converted to SAM specification 1.6 CRAM files with GATK 4.1.4.1 using the GATK Exome Best Practices Workflow (https://github.com/gatk-workflows/gatk4-exome-analysis-pipeline). Following quality control, 12,685 samples were ultimately retained^20^.

### Curation of rare gene variants for CMP

To identify protein-altering coding variants, sequencing data were annotated using the Ensembl Variant Effect Predictor (v105)^21^ and the Loss-of-Function Transcript Effect Estimator (LOFTEE)^22^ plug-in (https://github.com/konradjk/loftee). Protein-truncating variants were identified as LOFTEE high-confidence variants, excluding any variant with a flag and restricting to variants affecting the canonical Ensembl transcript of a given gene. Variants were also annotated using the ClinVar database^23^ (updated in April 2023), restricting to variants with an entry from 2015 or later submitted from a clinical testing laboratory. The submitting laboratory had to indicate that assertion criteria were provided and that American College of Medical Genetics and Genomics and Association for Molecular Pathology guidelines informed the classification for consideration in our study.

Further restriction to protein-coding variants with a ‘likely pathogenic’ or ‘pathogenic’ classification (P/LP) was used for the present analysis, excluding variants with a conflicting assertion in ClinVar.

These variant annotations were used to identify carriers of disease-causing variation for CMP. Specifically, we used ClinVar P/LP variants restricting to those affecting genes with at least strong evidence for a specific heritable CMP (DCM^24^, HCM^25^ or ARVC^26^), taking into account the phenotypes for which the ClinVar variants were reported. Protein-truncating variants were included for select genes, depending on known gene–disease mechanisms^27^.

Carriers of disease-causing variants were defined for DCM as individuals carrying ClinVar P/LP variants—if reported for DCM—in *BAG3*, *DES*, *TNNT2*, *FLNC*, *PLN*, *LMNA*, *MYH7*, *RBM20*, *SCN5A*, *TNNC1* or *DSP*; or individuals carrying protein-truncating variants in *BAG3*, *FLNC*, *LMNA* or *DSP*; or individuals carrying truncating variants in cardiac-expressed exons (percent spliced in >90%) of *TTN*^28,29^. Carriers of disease-causing variants were defined for HCM as individuals carrying ClinVar P/LP variants—if reported for HCM—in *MYH7*, *MYBPC3*, *TNNT2*, *TNNI3*, *TPM1*, *MYL2*, *MYL3*, *ACTC1*, *ACTN2*, *CSRP3*, *PLN*, *TTR* or *PRKAG2*; or individuals carrying protein-truncating variants in MYBPC3 or PLN. Carriers of disease-causing variants were defined for ARVC as individuals carrying ClinVar P/LP variants—if reported for ARVC—in *PKP2*, *DSP*, *TMEM43*, *DSC2* or *DSG2*; or individuals carrying protein-truncating variants in *PKP2*, *DSP*, *DSC2* or *DSG2*. While assignment to specific CMP subphenotypes (DCM, HCM and ARVC) required the ClinVar entry to explicitly report the respective phenotype, the omnibus CMP category included all P/LP variants in the curated gene list, regardless of the specific phenotype reported, to capture the aggregate burden of pathogenic variation. Individuals with variants associated with multiple phenotypes were counted within each respective subtype but only once in the composite analysis.

We note that the above curation might miss pathogenic nontruncating variants if those are not present (or did not pass our strict filters) in ClinVar. Nevertheless, we posit that the pipeline is specific for disease-causing variation, as indicated by marked effect sizes for HCM in previous work^27^.

### Study outcomes

The primary endpoint specified for this analysis was HHF. All events were adjudicated by a central clinical events committee blinded to treatment assignment^2,30^. HHF was selected as the primary outcome based on the established efficacy profile of dapagliflozin in DECLARE-TIMI 58, where clinical benefit was driven by reduction in HHF events rather than atherothrombotic endpoints, as well as the specific phenotypic relevance of HF to CMP variant carriers. Secondary exploratory endpoints included the composite of CV death or HHF, isolated CV death and all-cause mortality.

### Statistical analysis

Cox proportional hazard models were performed to calculate HRs for carriers versus noncarriers and incident HHF, as well as for dapagliflozin versus placebo by CMP carrier status (including subcategories of DCM, HCM, ARVC and *TTN*) to estimate efficacy to prevent HHF. The association of CMP variant carrier status and incident HHF was performed for the placebo arm only and adjusted for age, sex, body mass index, current smokers at baseline, systolic blood pressure, history of coronary artery disease, prior myocardial infarction, estimated glomerular filtration rate and the principal components 1–10 for genetic ancestry. A model including adjustment for baseline log(NT-proBNP) values was also explored. Because the comparison of dapagliflozin versus placebo in CMP carriers did not break randomization, we adjusted these analyses by age, sex and principal components 1–10 for genetic ancestry. Further adjusting by history of prior myocardial infarction and log(NT-proBNP) values was also explored as a sensitivity analysis. In addition, outcomes were stratified by a history of HF. We tested the proportional hazards assumption using Schoenfeld residuals and found no violations. Kaplan–Meier survival curves were generated for each incident outcome stratified by carrier status. Treatment interaction between dapagliflozin and CMP mutation was assessed using a Cox model. Analyses were performed on an available-case basis; no imputation methods were required due to minimal missing data. *P* values <0.05 were considered statistically significant. All analyses were performed using R version 4.4.2 (R Foundation).

### Reporting summary

Further information on research design is available in the Nature Portfolio Reporting Summary linked to this article.

## Online content

Any methods, additional references, Nature Portfolio reporting summaries, source data, extended data, supplementary information, acknowledgements, peer review information; details of author contributions and competing interests; and statements of data and code availability are available at 10.1038/s41591-026-04439-x.

## Supplementary information

Supplementary Information Supplementary Tables 1–6.

Reporting Summary

## Data Availability

Due to contractual agreements with sponsors, trial data are not available to share.

## Extended data

**Extended Data Table 1:** Baseline characteristics of cardiomyopathy variant carriers stratified by randomized treatment

	Placebo	Dapagliflozin
N	56	65
Age, years	63.1 ± 5.8	63.7 ± 6.8
Male sex	33 (59)	41 (63)
Ethnicity	Asian 2 (4)	Asian 5 (8)
	Black 1 (2)	Black 0 (0)
	Other 5 (9)	Other 5 (8)
	White 48 (86)	White 55 (85)
Body mass index, kg/m2	32.3 ± 4.9	32 ± 5.9
ASCVD	16 (29)	34 (52)
Prior myocardial infarction	6 (11)	17 (26)
History of hypertension	51 (91)	58 (89)
History of heart failure	9 (16)	13 (20)
History of chronic kidney disease	4 (7)	7 (11)
History of atrial fibrillation	3 (5)	11 (17)
Current smoker	7 (13)	9 (14)
Systolic blood pressure, mmHg	135.2 ± 15.9	132.5 ± 16.9
eGFR, mL/min/1.73m^2^	89.8 ± 22.9	85.9 ± 22.6
NT-proBNP pg/mL	284.2 ± 724.6	313.2 ± 527.7
hs-TnT ng/L	12 ± 7.8	14.9 ± 10.8
LVEF (%)	49.6 ± 14.3	51.8 ± 9.7
LVEF < 50%	8/16 (50%)	4/20 (20%)
LVEF < 40%	4/16 (25%)	3/20 (15%)
MRA therapy	5 (8.9%)	10 (15.4%)
RAAS inhibitors therapy	48 (85.7%)	50 (76.9%)
DCM variant carriers	37 (66)	39 (60)
*TTN* carriers	29 (52)	28 (43)
HCM variant carriers	14 (25)	11 (17)
ARVC variant carriers	7 (13)	18 (28)

Baseline characteristics of carriers of cardiomyopathy-associated variants randomized to either dapagliflozin treatment or placebo. Continuous variables are reported as mean ± SD, categorical variables as n(%). ASCVD: atherosclerotic cardiovascular disease; eGFR: estimated glomerular filtration rate; NT-proBNP: N-terminal pro-brain natriuretic peptide; hs-TnT: high-sensitivity cardiac troponin T; RAAS: renin-angiotensin-aldosterone system; LVEF: left ventricular ejection fraction; DCM: dilated cardiomyopathy; HCM: hypertrophic cardiomyopathy; TTN: Titin gene; ARVC: arrhythmogenic right ventricular cardiomyopathy.

**Extended Data Table 2:** Baseline characteristics in cardiomyopathy variant carriers stratified by history of heart failure

Characteristic	Carriers of CMP-associated variants History of HF N = 22	Carriers of CMP-associated variants No History of HF N = 99	Non-carriers History of HF N = 1,235	Non-carriers No History of HF N = 11,329	p-value
Age	64.2 ± 7	63.3 ± 6.2	64.4 ± 7.4	63.9 ± 6.8	0.075
Male sex	17 (77.3%)	57 (57.6%)	795 (64.4%)	7,256 (64%)	0.317
Ethnicity	Asian 0 (0.0%) Black 0 (0.0%) Other 0 (0.0%) White 22 (100.0%)	Asian 7 (7.1%) Black 1 (1.0%) Other 10 (10.1%) White 81 (81.8%)	Asian 36 (2.9%) Black 42 (3.4%) Other 23 (1.9%) White 1134 (91.8%)	Asian 1,104 (9.7%) Black 354 (3.1%) Other 419 (3.7%) White 9,452 (83.4%)	1.23e-16
BMI (kg/m2)	34.4 ± 6.1	31.6 ± 5.2	34.2 ± 6.3	32.3 ± 6	3.69e-24
ASCVD	14 (63.6%)	36 (36.4%)	831 (67.3%)	4,434 (39.1%)	1.9e-79
Prior myocardial infarction	6 (27.3%)	17 (17.2%)	560 (45.3%)	2,205 (19.5%)	2.5e-94
History of hypertension	21 (95.5%)	88 (88.9%)	1,158 (93.8%)	10,128 (89.4%)	2.38e-05
History of chronic kidney disease	1 (4.5%)	10 (10.1%)	172 (13.9%)	772 (6.8%)	1.06e-17
History of atrial fibrillation	6 (27.3%)	8 (8.1%)	284 (23%)	583 (5.1%)	9.06e-122
Current smoker	5 (22.7%)	11 (11.1%)	169 (13.7%)	1,601 (14.1%)	0.519
Systolic blood pressure	129.7 ± 16.4	134.7 ± 16.4	134.6 ± 15.7	135.1 ± 15.3	0.470
eGFR (mL/min/1.73m2)	83.8 ± 18	88.5 ± 23.6	80.7 ± 22.1	86.4 ± 21.6	3.53e-18
NT-proBNP pg/mL	629 ± 722.5	234.4 ± 586.6	445.8 ± 614.5	141 ± 253.6	2.05e-184
hs-TnT ng/L	18.3 ± 9.5	12.6 ± 9.4	18 ± 21.4	12.6 ± 14.6	2.81e-70
LVEF (%) [N=3,247]	43.9 ± 13.8	54.2 ± 9.3	48.8 ± 12.9	58.8 ± 9.4	4.78e-81
LVEF < 50%	7/12 (58.3%)	5/24 (20.8%)	345/744 (46.4%)	322/2,467 (13.1%)	4.67e-85
LVEF < 40%	5/12 (41.7%)	2/24 (8.3%)	174/744 (23.4%)	66/2,467 (2.7%)	1.27e-79
MRA therapy	9 (40.9%)	6 (6.1%)	268 (21.7%)	324 (2.9%)	1.59e-201
RAAS inhibitors therapy	19 (86.4%)	79 (79.8%)	1,068 (86.5%)	9,314 (82.2%)	0.002

Continuous variables are reported as mean ± SD and tested with a 2-sided Wilcoxon rank-sum test. Categorical variables are reported as n(%) and tested with a 2-sided chi-squared test. ASCVD: atherosclerotic cardiovascular disease; eGFR: estimated glomerular filtration rate; NT-proBNP: N-terminal pro-brain natriuretic peptide; hs-TnT: high-sensitivity cardiac troponin T; MRA: mineralocorticoid receptor antagonist; RAAS: renin-angiotensin-aldosterone system; LVEF: left ventricular ejection fraction.
