# Supplementary material for: Effects of SGLT2 inhibition on incident heart failure in carriers of cardiomyopathy-associated genetic variants
Source: Nat Med. 2026 Jun 8;32(6):2288–93. doi: 10.1038/s41591-026-04439-x (PMC13278951; doi:10.1038/s41591-026-04439-x)
Supplement: Supplementary file 1 — Supplementary Tables 1–6. [file 41591_2026_4439_MOESM1_ESM.pdf]

nature medicine

Supplementary information https://doi.org/10.1038/s41591-026-04439-x

Effects of SGLT2 inhibition on incident heart failure in carriers of cardiomyopathy-associated genetic variants

In the format provided by the authors and unedited

=

Table of Contents

Supplementary Tables 2

Supplementary Table 1: Baseline Characteristics of the genetic cohort compared with the overall DECLARE cohort.. 2

Supplementary Table 2: Outcomes based on other DECLARE-TIMI efficacy outcomes 1

Supplementary Table 3: Hospitalization for heart failure stratified by cardiomyopathy variant, carrier status, and treatment assignment 2

Supplementary Table 4: Hospitalization for heart failure stratified by cardiomyopathy variant, carrier status, and treatment assignment adjusted for baseline History of myocardial infarction and NT-proBNP 3

Supplementary Table 5: Hospitalization for heart failure stratified by cardiomyopathy variant, carrier status, and treatment assignment among patients without a history of heart failure 5

Supplementary Table 6: Hospitalization for heart failure stratified by cardiomyopathy variant, carrier status, and treatment assignment among patients with a history of heart failure 6

Supplementary Tables

Supplementary Table 1: Baseline Characteristics of the genetic cohort compared with the overall DECLARE cohort

Characteristic	Genetic Cohort N = 12,685	Overall Trial Cohort N = 17,160
Age, years	64 ± 6.9	63.9 ± 6.8
Male sex	8,125 (64.1%)	10,738 (62.6%)
Ethnicity	Asian 1,147 (9.0%)	Asian 2,303 (13.4%)
	Black 397 (3.1%)	Black 603 (3.5%)
	Other 452 (3.6%)	Other 601 (3.5%)
	White 10,689 (84.3%)	White 13,653 (79.6%)
Body mass index, kg/m^2	32.5 ± 6	32.1 ± 6
ASCVD	5,315 (41.9%)	6,931 (40.4%)
Prior myocardial infarction	2,788 (22%)	3,584 (20.9%)
History of hypertension	11,395 (89.8%)	15,427 (89.9%)
History of heart failure	1,257 (9.9%)	1,724 (10%)
History of chronic kidney disease	955 (7.5%)	1,265 (7.4%)
History of atrial fibrillation	881 (6.9%)	1,116 (6.5%)
Current smoker	1,786 (14.1%)	2,498 (14.6%)
Systolic blood pressure, mmHg	135.1 ± 15.3	135 ± 15.4
eGFR, mL/min/1.73m^2	85.9 ± 21.7	86.1 ± 21.8
NT-proBNP pg/mL	172.2 ± 325.8	172.7 ± 347
hs-TnT ng/L	13.1 ± 15.4	13 ± 14.7
LVEF (%)	56.4 ± 11.2	56.5 ± 11.3
LVEF < 50%	679 (20.9%)	864 (21.1%)
LVEF < 40%	247 (7.6%)	313 (7.7%)
MRA therapy	607 (4.8%)	762 (4.4%)
RAAS inhibitors therapy	10,480 (82.6%)	13,962 (81.4%)

Baseline characteristics of genetic cohort and the overall cohort of the DECLARE-TIMI 58 trial. Presented as counts (percentage) or mean ± standard deviation. ASCVD: atherosclerotic cardiovascular disease; eGFR: estimated glomerular filtration rate; NT-proBNP: N-terminal pro-brain natriuretic peptide; hs-TnT: high-sensitivity cardiac troponin T; MRA: mineralocorticoid receptor antagonist; RAAS: renin-angiotensin-aldosterone system; LVEF: left ventricular ejection fraction

Supplementary Table 2: Outcomes based on other DECLARE-TIMI efficacy outcomes

Outcome	Mutation	Treatment	N events/N total	Event Rate %	Adjusted Hazard Ratio (95% CI)	p-Int (HR)	Absolute Risk Reduction (95% CI)	p-Int (AR)
CV death	Carrier	Placebo	0/56	0%	NA	0.988	-3.1% (-7.3,1.2)	0.144
	Carrier	Dapagliflozin	2/65	3.1%				
	Non-carrier	Placebo	186/6,291	3%	0.96 (0.78,1.18)		0.1% (-0.5,0.7)	
	Non-carrier	Dapagliflozin	179/6,273	2.9%				
CV death or HHF	Carrier	Placebo	9/56	16.1%	0.38 (0.12,1.27)	0.0737	9.9% (-1.4,21.3)	0.13
	Carrier	Dapagliflozin	4/65	6.2%				
	Non-carrier	Placebo	375/6,291	6%	0.80 (0.69,0.93)		1.1% (0.3,1.9)	
	Non-carrier	Dapagliflozin	304/6,273	4.8%				
All-cause death	Carrier	Placebo	4/56	7.1%	1.05 (0.26,4.27)	0.802	1% (-8,10)	0.911
	Carrier	Dapagliflozin	4/65	6.2%				
	Non-carrier	Placebo	422/6,291	6.7%	0.92 (0.80,1.06)		0.5% (-0.4,1.3)	
	Non-carrier	Dapagliflozin	391/6,273	6.2%				

CV: Cardiovascular; HHF: Hospitalization for heart failure

Supplementary Table 3: Hospitalization for heart failure stratified by cardiomyopathy variant, carrier status, and treatment assignment

P/LP variants	Mutation	Treatment	N events/N total	Event Rate %	Adjusted Hazard Ratio (95% CI)	Absolute Risk Reduction (95% CI)
DCM	Carrier	Placebo	5/37	13.5%	0.19 (0.02 - 1.70)	10.9% (-1.3 - 23.2)
DCM	Carrier	Dapagliflozin	1/39	2.6%		
DCM	Non-carrier	Placebo	225/6,310	3.6%	0.69 (0.56 - 0.85)	1.1% (0.5 - 1.7)
DCM	Non-carrier	Dapagliflozin	158/6,299	2.5%		
TTN	Carrier	Placebo	5/29	17.2%	0.19 (0.02 - 1.68)	13.7% (-2 - 29.3)
TTN	Carrier	Dapagliflozin	1/28	3.6%		
TTN	Non-carrier	Placebo	225/6,318	3.6%	0.69 (0.56 - 0.85)	1.1% (0.5 - 1.7)
TTN	Non-carrier	Dapagliflozin	158/6,310	2.5%		
HCM	Carrier	Placebo	3/14	21.4%	0.53 (0.05 - 5.10)	12.3% (-16.2 - 40.9)
HCM	Carrier	Dapagliflozin	1/11	9.1%		
HCM	Non-carrier	Placebo	227/6,333	3.6%	0.68 (0.56 - 0.84)	1.1% (0.5 - 1.7)
HCM	Non-carrier	Dapagliflozin	158/6,327	2.5%		
ARVC	Carrier	Placebo	0/7	0%	NA	0% (0 - 0)
ARVC	Carrier	Dapagliflozin	0/18	0%		
ARVC	Non-carrier	Placebo	230/6,340	3.6%	0.68 (0.56 - 0.83)	1.1% (0.5 - 1.7)
ARVC	Non-carrier	Dapagliflozin	159/6,320	2.5%		

Detailed overview of hospitalization for heart failure outcomes provided for each carrier group and stratified by treatment group. P/LP: pathogenic/likely pathogenic; DCM: dilated cardiomyopathy; *TTN*: Titin gene; HCM: hypertrophic cardiomyopathy; ARVC: arrhythmogenic right ventricular cardiomyopathy; CI: confidence interval

Supplementary Table 4: Hospitalization for heart failure stratified by cardiomyopathy variant, carrier status, and treatment assignment adjusted for baseline History of myocardial infarction and NT-proBNP

P/LP Variants	Mutation	Treatment	N events/N total	Event Rate %	Adjusted Hazard Ratio (95% CI)	Absolute Risk Reduction (95% CI)	p-Int (HR)	p-Int (AR)
CMP	Carrier	Placebo	9/56	16.1%	0.06 (0.01,0.49)	13% (2.4,23.6)	0.00991	0.027
CMP	Carrier	Dapagliflozin	2/65	3.1%				
CMP	Non-carrier	Placebo	221/6,291	3.5%	0.71 (0.58,0.88)	1% (0.4,1.6)		
CMP	Non-carrier	Dapagliflozin	157/6,273	2.5%				
DCM	Carrier	Placebo	5/37	13.5%	0.08 (0.01,0.98)	10.9% (-1.3,23.2)	0.0609	0.114
DCM	Carrier	Dapagliflozin	1/39	2.6%				
DCM	Non-carrier	Placebo	225/6,310	3.6%	0.70 (0.57,0.86)	1.1% (0.5,1.7)		
DCM	Non-carrier	Dapagliflozin	158/6,299	2.5%				
TTN	Carrier	Placebo	5/29	17.2%	0.09 (0.01,1.15)	13.7% (-2,29.3)	0.0971	0.114
TTN	Carrier	Dapagliflozin	1/28	3.6%				
TTN	Non-carrier	Placebo	225/6,318	3.6%	0.70 (0.56,0.86)	1.1% (0.5,1.7)		
TTN	Non-carrier	Dapagliflozin	158/6,310	2.5%				
HCM	Carrier	Placebo	3/14	21.4%	NA	12.3% (-16.2,40.9)	0.987	0.44
HCM	Carrier	Dapagliflozin	1/11	9.1%				

HCM	Non-carrier	Placebo	227/6,333	3.6%	0.69 (0.56,0.85)	1.1% (0.5,1.7)		
HCM	Non-carrier	Dapagliflozin	158/6,327	2.5%				
ARVC	Carrier	Placebo	0/7	0%	NA	0% (0,0)	1	NA
ARVC	Carrier	Dapagliflozin	0/18	0%				
ARVC	Non-carrier	Placebo	230/6,340	3.6%	0.68 (0.55,0.84)	1.1% (0.5,1.7)		
ARVC	Non-carrier	Dapagliflozin	159/6,320	2.5%				

Detailed overview of hospitalization for heart failure outcomes provided for each carrier group and stratified by treatment group and additionally adjusted for history of myocardial infarction and baseline NT-proBNP levels. P/LP: pathogenic/likely pathogenic; DCM: dilated cardiomyopathy; *TTN*: Titin gene; HCM: hypertrophic cardiomyopathy; ARVC: arrhythmogenic right ventricular cardiomyopathy; CI: confidence interval

Supplementary Table 5: Hospitalization for heart failure stratified by cardiomyopathy variant, carrier status, and treatment assignment among patients without a history of heart failure

P/LP variants	Mutation	Treatment	N events/N total	Event Rate %	Adjusted Hazard Ratio (95% CI)	Absolute Risk Reduction (95% CI)
DCM	Carrier	Placebo	2/30	6.7%	NA	6.7% (-2.4 - 15.7)
DCM	Carrier	Dapagliflozin	0/32	0%		
DCM	Non-carrier	Placebo	130/5,676	2.3%	0.69 (0.53 - 0.91)	0.7% (0.2 - 1.2)
DCM	Non-carrier	Dapagliflozin	91/5,690	1.6%		
TTN	Carrier	Placebo	2/24	8.3%	NA	8.3% (-3 - 19.6)
TTN	Carrier	Dapagliflozin	0/21	0%		
TTN	Non-carrier	Placebo	130/5,682	2.3%	0.69 (0.53 - 0.90)	0.7% (0.2 - 1.2)
TTN	Non-carrier	Dapagliflozin	91/5,701	1.6%		
HCM	Carrier	Placebo	3/12	25%	NA	25% (-0.6 - 50.6)
HCM	Carrier	Dapagliflozin	0/9	0%		
HCM	Non-carrier	Placebo	129/5,694	2.3%	0.70 (0.53 - 0.91)	0.7% (0.2 - 1.2)
HCM	Non-carrier	Dapagliflozin	91/5,713	1.6%		
ARVC	Carrier	Placebo	0/6	0%	NA	0% (0 - 0)
ARVC	Carrier	Dapagliflozin	0/15	0%		
ARVC	Non-carrier	Placebo	132/5,700	2.3%	0.68 (0.52 - 0.89)	0.7% (0.2 - 1.2)
ARVC	Non-carrier	Dapagliflozin	91/5,707	1.6%		

Detailed overview of hospitalization for heart failure outcomes provided for each carrier group and stratified by treatment group in patients without a history of heart failure. P/LP: pathogenic/likely pathogenic; DCM: dilated cardiomyopathy; *TTN*: Titin gene; HCM: hypertrophic cardiomyopathy; ARVC: arrhythmogenic right ventricular cardiomyopathy; CI: confidence interval

Supplementary Table 6: Hospitalization for heart failure stratified by cardiomyopathy variant, carrier status, and treatment assignment among patients with a history of heart failure

P/LP variants	Mutation	Treatment	N events/ N total	Event Rate %	Adjusted Hazard Ratio (95% CI)	Absolute Risk Reduction (95% CI)
DCM	Carrier	Placebo	3/7	42.9%	0.37 (0.04 - 3.69)	28.6% (-19.9 - 77.1)
DCM	Carrier	Dapagliflozin	1/7	14.3%		
DCM	Non-carrier	Placebo	95/634	15%	0.68 (0.49 - 0.93)	4% (0.3 - 7.7)
DCM	Non-carrier	Dapagliflozin	67/609	11%		
TTN	Carrier	Placebo	3/5	60%	0.23 (0.02 - 2.34)	45.7% (-9.9 - 101.3)
TTN	Carrier	Dapagliflozin	1/7	14.3%		
TTN	Non-carrier	Placebo	95/636	14.9%	0.68 (0.50 - 0.93)	3.9% (0.2 - 7.7)
TTN	Non-carrier	Dapagliflozin	67/609	11%		
HCM	Carrier	Placebo	0/2	0%	NA	-50% (-148 - 48)
HCM	Carrier	Dapagliflozin	1/2	50%		
HCM	Non-carrier	Placebo	98/639	15.3%	0.65 (0.48 - 0.89)	4.4% (0.7 - 8.2)
HCM	Non-carrier	Dapagliflozin	67/614	10.9%		
ARVC	Carrier	Placebo	0/1	0%	NA	NA
ARVC	Carrier	Dapagliflozin	0/3	0%		
ARVC	Non-carrier	Placebo	98/640	15.3%	0.67 (0.49 - 0.91)	4.2% (0.5 - 8)
ARVC	Non-carrier	Dapagliflozin	68/613	11.1%		

Detailed overview of hospitalization for heart failure outcomes provided for each carrier group and stratified by treatment group in patients with a history of heart failure. P/LP: pathogenic/likely pathogenic; DCM: dilated cardiomyopathy; *TTN*: Titin gene; HCM: hypertrophic cardiomyopathy; ARVC: arrhythmogenic right ventricular cardiomyopathy; CI: confidence interval
