# Supplementary material for: Effects of SGLT2 inhibition on incident heart failure in carriers of cardiomyopathy-associated genetic variants
Source: Nat Med. 2026 Jun 8;32(6):2288–93. doi: 10.1038/s41591-026-04439-x (PMC13278951; doi:10.1038/s41591-026-04439-x)
Supplement: Supplementary file 2 — Reporting Summary [file 41591_2026_4439_MOESM2_ESM.pdf]

nature portfolio

Corresponding author(s): Shinwan Kany

Last updated by author(s): Apr 28, 2026

Reporting Summary

Nature Portfolio wishes to improve the reproducibility of the work that we publish. This form provides structure for consistency and transparency in reporting. For further information on Nature Portfolio policies, see our Editorial Policies and the Editorial Policy Checklist.

Statistics

For all statistical analyses, confirm that the following items are present in the figure legend, table legend, main text, or Methods section.

n/a	Confirmed	
☐	☒	The exact sample size (*n*) for each experimental group/condition, given as a discrete number and unit of measurement
☐	☒	A statement on whether measurements were taken from distinct samples or whether the same sample was measured repeatedly
☐	☒	The statistical test(s) used AND whether they are one- or two-sided<br>*Only common tests should be described solely by name; describe more complex techniques in the Methods section.*
☐	☒	A description of all covariates tested
☐	☒	A description of any assumptions or corrections, such as tests of normality and adjustment for multiple comparisons
☐	☒	A full description of the statistical parameters including central tendency (e.g. means) or other basic estimates (e.g. regression coefficient) AND variation (e.g. standard deviation) or associated estimates of uncertainty (e.g. confidence intervals)
☐	☒	For null hypothesis testing, the test statistic (e.g. *F*, *t*, *r*) with confidence intervals, effect sizes, degrees of freedom and *P* value noted<br>*Give P values as exact values whenever suitable.*
☒	☐	For Bayesian analysis, information on the choice of priors and Markov chain Monte Carlo settings
☒	☐	For hierarchical and complex designs, identification of the appropriate level for tests and full reporting of outcomes
☐	☒	Estimates of effect sizes (e.g. Cohen's *d*, Pearson's *r*), indicating how they were calculated

Our web collection on statistics for biologists contains articles on many of the points above.

Software and code

Policy information about availability of computer code

Data collection	No software was used for data collection.
Data analysis	Sequencing data was annotated using the Ensembl Variant Effect Predictor (VEP; v105) and the Loss-of-Function Transcript Effect Estimator (LOFTEE) plug-in (https://github.com/konradjk/loftee). All analyses were performed using R version 4.4.2 (R Foundation).

For manuscripts utilizing custom algorithms or software that are central to the research but not yet described in published literature, software must be made available to editors and reviewers. We strongly encourage code deposition in a community repository (e.g. GitHub). See the Nature Portfolio guidelines for submitting code & software for further information.

Data

Policy information about availability of data

All manuscripts must include a data availability statement. This statement should provide the following information, where applicable:

- Accession codes, unique identifiers, or web links for publicly available datasets
- A description of any restrictions on data availability
- For clinical datasets or third party data, please ensure that the statement adheres to our policy

Because of contractual agreements with sponsors, trial data are not available to share.

Research involving human participants, their data, or biological material

Policy information about studies with human participants or human data. See also policy information about sex, gender (identity/presentation), and sexual orientation and race, ethnicity and racism.

Reporting on sex and gender	We report sex in in Table 1 and Extended Data Tables 1-2, Supplementary Table 1 and adjust for it in our analysis. Our data was not powered for sex-specific analysis due to the number of variant carriers.
Reporting on race, ethnicity, or other socially relevant groupings	We report ethnicity in in Table 1 and Extended Data Tables 1-2, Supplementary Table 1
Population characteristics	Population characteristics are outlined in Table 1 and Extended Data Tables 1-2, Supplementary Table 1
Recruitment	Patients were recruited in DECLARE as outlined in the original publication and trial protocol (DOI: 10.1056/NEJMoa1812389). We discuss potential biases from selection due to age and cardiovascular burden in the discussion/limitation.
Ethics oversight	Each country and site had ethical oversight through local and/or central IRBs.

Note that full information on the approval of the study protocol must also be provided in the manuscript.

Field-specific reporting

Please select the one below that is the best fit for your research. If you are not sure, read the appropriate sections before making your selection.

☒ Life sciences ☐ Behavioural & social sciences ☐ Ecological, evolutionary & environmental sciences

For a reference copy of the document with all sections, see nature.com/documents/nr-reporting-summary-flat.pdf

Life sciences study design

All studies must disclose on these points even when the disclosure is negative.

Sample size	The sample size was determined by the available rare cardiomyopathy-associated variant carriers. We observed a prevalence comparable with the population prevalence expected.
Data exclusions	Trial participants who did not consent for sequencing and those with samples not passing genetic QC were excluded.
Replication	No replication cohort with a SGLT2i random group was available to us.
Randomization	Within DECLARE, random allocation of therapy was used. Since genetic variants were not known to participants or investigators, we do not expect randomization to break.
Blinding	DECLARE was a double-blind, placebo-controlled trial were both patient and investigator were blinded to treatment assignment Endpoints were adjudicated by a centralized, blinded clinical endpoint committee. As outlined above, since sequencing was performed after the trial, variant carrier status was unknown to patients and investigators.

Reporting for specific materials, systems and methods

We require information from authors about some types of materials, experimental systems and methods used in many studies. Here, indicate whether each material, system or method listed is relevant to your study. If you are not sure if a list item applies to your research, read the appropriate section before selecting a response.

Materials & experimental systems

n/a	Involved in the study	
☒	☐	Antibodies
☒	☐	Eukaryotic cell lines
☒	☐	Palaeontology and archaeology
☒	☐	Animals and other organisms
☐	☒	Clinical data
☒	☐	Dual use research of concern
☒	☐	Plants

Methods

n/a	Involved in the study	
☒	☐	ChIP-seq
☒	☐	Flow cytometry
☒	☐	MRI-based neuroimaging

Clinical data

Policy information about clinical studies

All manuscripts should comply with the ICMJE guidelines for publication of clinical research and a completed CONSORT checklist must be included with all submissions.

Clinical trial registration	NCT01730534
Study protocol	Attached in the original publication (DOI: 10.1056/NEJMoa1812389).
Data collection	Clinical data was collected prospectively at the site level and entered into a central database for each trial. DECLARE collected data from 2013-2018.
Outcomes	Primary and secondary endpoints for each trial were pre-specified in the trial proptocols and statistical analysis plan. They were collected by sites and sent to the CEC for blinded adjudication based on the CEC Charter for each trial.

Plants

Seed stocks	*Report on the source of all seed stocks or other plant material used. If applicable, state the seed stock centre and catalogue number. If plant specimens were collected from the field, describe the collection location, date and sampling procedures.*
Novel plant genotypes	*Describe the methods by which all novel plant genotypes were produced. This includes those generated by transgenic approaches, gene editing, chemical/radiation-based mutagenesis and hybridization. For transgenic lines, describe the transformation method, the number of independent lines analyzed and the generation upon which experiments were performed. For gene-edited lines, describe the editor used, the endogenous sequence targeted for editing, the targeting guide RNA sequence (if applicable) and how the editor was applied.*
Authentication	*Describe any authentication procedures for each seed stock used or novel genotype generated. Describe any experiments used to assess the effect of a mutation and, where applicable, how potential secondary effects (e.g. second site T-DNA insertions, mosiacism, off-target gene editing) were examined.*
